# Immunological characteristics in elderly COVID-19 patients: a post-COVID era analysis

**DOI:** 10.3389/fcimb.2024.1450196

**Published:** 2024-11-29

**Authors:** Yunhui Li, Yuan Chen, Jing Liang, Yajie Wang

**Affiliations:** Department of Clinical Laboratory, Beijing Ditan Hospital, Capital Medical University, Beijing, China

**Keywords:** COVID-19, immunological characteristics, the elderly, Cd4 + T cell, gene set enrichment analysis

## Abstract

**Background:**

Advanced age is a primary risk factor for adverse COVID-19 outcomes, potentially attributed to immunosenescence and dysregulated inflammatory responses. In the post-pandemic era, with containment measures lifted, the elderly remain particularly susceptible, highlighting the need for intensified focus on immune health management.

**Methods:**

A total of 281 elderly patients were enrolled in this study and categorized based on their clinical status at the time of admission into three groups: non-severe (n = 212), severe survivors (n = 49), and severe non-survivors (n = 20). Binary logistic regression analysis was employed to identify independent risk factors associated with disease severity and in-hospital outcomes. The diagnostic performance of risk factors was assessed using the receiver operating characteristic (ROC) curves. Kaplan-Meier survival analysis and log-rank test were utilized to compare the 30-day survival rates. Furthermore, the transcriptomic data of CD4^+^ T cells were extracted from Gene Expression Omnibus (GEO) database. Gene Set Enrichment Analysis (GSEA) was applied to reveal biological processes and pathways involved.

**Results:**

In the comparison between severe and non-severe COVID-19 cases, significant elevations were observed in the neutrophil-to-lymphocyte ratio (NLR), C-reactive protein (CRP), and Serum Amyloid A (SAA) levels, concurrent with a notable reduction in CD8^+^ T cells, CD4^+^ T cells, natural killer (NK) cells, and monocytes (all *p* < 0.05). CD4^+^ T cells (OR: 0.997 [0.995-1.000], *p*<0.05) and NLR (OR: 1.03 [1.001-1.060], *p*<0.05) were independent risk factors affecting disease severity. The diagnostic accuracy for COVID-19 severity, as measured by the area under the curve (AUC) for CD4^+^ T cells and NLR, was 0.715 (95% CI: 0.645-0.784) and 0.741 (95% CI: 0.675-0.807), respectively. Moreover, patients with elevated NLR or IL-6 levels at admission exhibited significantly shorter survival times. Gene Set Enrichment Analysis (GSEA) revealed several biological pathways that are implicated in the regulation of immune responses and metabolic processes.

**Conclusions:**

Lymphocytopenia and the cytokine storm onset are significant predictors of an unfavorable prognosis in elderly patients. The decrease in CD4^+^ T cells among elderly patients is detrimental to disease recovery, and the biological pathways regulated by these cells could potentially heighten vulnerability to SARS-CoV-2 infection, thereby exacerbating the development of associated complications.

## 1 Introduction

Since the emergence of the severe acute respiratory syndrome coronavirus 2 (SARS-CoV-2) in December 2019, this virus has rapidly spread globally, causing the COVID-19 pandemic. As of September 1, 2024, the contagion has resulted in over 770 million confirmed cases and more than 7 million deaths worldwide (https://covid19.who.int/), posing an unprecedented challenge to global public health.

COVID-19 patients exhibit differential disease severity after SARS-CoV-2 infection, from asymptomatic cases to critical ones, with a mortality rate that approaches 5% in severe cases. Innate and adaptive immunity are crucial for combating SARS-CoV-2 infection, involving intricate interactions among myeloid, lymphoid, and non-hematopoietic cellular components, as well as a diversity of molecular mediators that synergize to enhance host defense and facilitate viral clearance. Neutrophils, known for their ability to release neutrophil extracellular traps (NETs), have been observed at elevated levels in the serum and bronchoalveolar lavage fluid of patients with severe COVID-19 (Leppkes et al., 2020). The presence of NETs correlates with an increased mortality risk (Godement et al., 2021; Silva et al., 2023). Circulating monocytes tend to migrate into pulmonary tissue and differentiate into inflammatory macrophages, with elderly patients showing an increased proportion of pro-inflammatory monocyte subsets characterized by the CD14^+^CD16^+^ phenotype, which are linked to an amplified cytokine response (Nyugen et al., 2010). In COVID-19 patients with severe respiratory failure (SRF), either macrophage-activation syndrome or a marked reduction in human leukocyte antigen-D related (HLA-DR) expression, along with a profound depletion of CD4 lymphocytes, CD19 lymphocytes, and natural killer (NK) cells, has been observed (Giamarellos-Bourboulis et al., 2020). CD8^+^ T cells are crucial for limiting pathogen dissemination by eliminating virus-infected cells during natural infections. CD4^+^ T cells, serving as key activators of both CD8^+^ T cells and B cells, provide signals for the maturation of the humoral immune response. In some COVID-19 cases, a “cytokine storm” may ensue, representing a dysregulated hyperinflammatory response. This phenomenon, characterized by a rapid increase in cytokine levels and immune cell dysregulation, is a significant contributor to the increased mortality risk in patients with severe COVID-19 (Vanderbeke et al., 2021).

The emergence of COVID-19 pandemic has precipitated an acceleration in vaccine development and administration. The global vaccination initiative has been instrumental in curbing disease progression and preventing hospitalizations (Lopez et al., 2021). Nonetheless, a considerable proportion of the elderly, especially those aged 80 and above, have demonstrated significant hesitancy toward vaccination. Statistical data from 2022 indicated that the vaccination rates for primary series and booster doses among this demographic was 71.9% and 46.7%, respectively (Wang et al., 2023). In December 2022, concurrent with the adjustment of China’s public health strategies and the end of the dynamic “zero-COVID” policy, there was a notable escalation in hospitalization rates among the elderly, resulting in a significant surge in cases numbers (Xiao et al., 2023).The heightened vulnerability of elderly individuals to SARS-CoV-2 infections, which can be partially attributed to immunosenescence, has been identified as a critical factor contributing to the intensification of disease severity and the increase in mortality risks. Consequently, it is urgent to explore potential mechanisms that could cause adverse outcomes for the elderly in the context of future waves of similar pandemics. The focus of research on elderly COVID-19 patients should transition from solely preventing infection to implementing effective strategies for reducing the incidence and mortality of severe cases. This study analyzed the immune characteristics of elderly patients at admission to reveal the immune dysregulation induced by SARS-CoV-2 infection. These findings not only offer potential biomarkers for predicting disease progression, but also yield significant implications for understanding the immunological challenges encountered by the elderly in the post-pandemic era, thereby providing a scientific foundation for developing effective prevention and treatment strategies.

## 2 Materials and methods

### 2.1 Study population

This retrospective study analyzed COVID-19 patients admitted to Beijing Ditan Hospital, affiliated with Capital Medical University, from December 2022 to March 2024. Notably, shortly after lifting the dynamic zero-COVID policy in China, at least two waves of SARS-CoV-2 outbreaks occurred. The first wave, from December 2022 to February 2023, was caused by Omicron BA.5.2/BF.7 variant (Pan et al., 2023); the second wave spanned from April 2023 and July 2023 and was triggered by the XBB variant (Ouyang et al., 2023). Thereafter, infected cases increased rapidly due to JN.1, a descendant of BA.2.86, which was designated as a variant of interest on December 18, 2023 (Looi, 2023). Consequently, the viral strains involved in this study were predominantly derived from the Omicron lineage and its sub-lineages.

The inclusion and exclusion criteria for subjects were as follows: Inclusion criteria: 1) Age over 60 years; 2) Confirmation of SARS-CoV-2 infection via polymerase chain reaction (PCR) testing or antigen detection; 3) Detection of immune-related cells and factors were conducted on the first day of hospitalization. Exclusion criteria: 1) Age under 60 years; 2) Lack of immune-related cells and factors detection results on the day of admission. All patients included in this study were clinically classified according to the China National Health Commission Guidelines for Diagnosis and Treatment of SARS-CoV-2 infection (Trial Version 10). The classification included: 1) Mild: characteristic symptoms of upper respiratory tract infections, such as dry and sore throat, cough, and fever; 2) Moderate: (i) persistent high fever for >3 days, or (and) other symptoms such as cough and shortness of breath, etc., with respiratory rate (RR) <30 breaths/minute, and oxygen saturation > 93% at rest. (ii) radiological imaging reveals characteristic presentations of COVID-19 pneumonia; 3) Severe: adults who meet any of the following criteria, which cannot be attributed to causes other than SARS-CoV-2 infection: (i) dyspnea, with RR ≥ 30 breaths/minute; (ii) oxygen saturation ≤ 93% at rest; (iii) arterial partial pressure of oxygen (PaO2)/fraction of inspired oxygen (FiO2) ≤ 300 mmHg (1 mmHg = 0.133 kPa); (iv) progressive clinical symptoms, with radiological imaging showing a significant progression of the lung lesions by >50% within 24-48 hours. 4) Critical: respiratory failure requiring mechanical ventilation; or shock; or multi-organ dysfunction requiring intensive care unit (ICU) monitoring and treatment.

A total of 281 patients were enrolled in this study. According to their clinical status at admission, 69 patients exhibiting severe or critical COVID-19 symptoms were classified into the severe group, while 212 patients presenting with mild to moderate symptoms were categorized as non-severe group. Furthermore, considering the in-hospital outcomes, the severe group were subcategorized into severe survivors (n=49) and severe non-survivors (n=20). This study was approved by the Institutional Review Board of the Ethics Committee of Ditan Hospital (NO.DTEC-KY2022-052-02).

### 2.2 Data collection

We collected demographic and clinical data from the electronic medical records of patients, including the initial immune-related cell and factor assay results upon admission, length of hospital stay, and in-hospital outcomes. The immunological assay results were classified into three distinct groups: lymphocyte subsets, comprising CD4^+^ T cells, CD8^+^ T cells, NK cells, and B cells; peripheral blood indicators, including monocyte and neutrophil-to-lymphocyte ratio (NLR); and inflammatory markers, such as Interleukin-6 (IL-6), C-reactive protein (CRP), and Serum Amyloid A (SAA).

Lymphocyte subset analysis was performed using reagents and equipment from BD Biosciences, with flow cytometry conducted on a BD FACSCanto™ II instrument. Complete blood count assessments were carried out utilizing an automated hematology analyzer (Sysmex XT-5000, Japan). IL-6 levels were measured via an automated electrochemiluminescence immunoassay system (Roche Cobas 8000 e801, Switzerland). SAA and CRP concentrations were determined with an automated biochemical analyzer (HITACHI 7600-020, Japan). Adherence to the manufacturers’ protocols was maintained throughout all experimental procedures.

### 2.3 Acquisition and processing of single-cell sequencing data

A comprehensive search of the Gene Expression Omnibus (GEO) database (https://www.ncbi.nlm.nih.gov/geo/) was conducted to obtain a dataset for elderly COVID-19 patients, employing screening criteria that encompassed both severe and non-severe cases. The RNA-sequencing data used for analysis in this study was extracted from the GSE165080 dataset, as reported by Zhang C et al (Bai et al., 2022; Ma et al., 2022; Wang et al., 2022). The dataset includes profiles of peripheral blood mononuclear cells (PBMCs) from healthy controls (HC), asymptomatic individuals (AS), and symptomatic COVID-19 patients (SM), capturing a spectrum of clinical features and disease severity levels.

### 2.4 Gene Set Enrichment Analysis

The transcriptomic data of CD4^+^ T cells were extracted from the GSE165080 dataset, followed by a comparative analysis of differential gene expression between severe and non-severe cases in elderly COVID-19 patients. To further investigate the potential mechanisms associated with the differentially expressed genes (DEGs) in this demographic, we retrieved the “c2.cp.kegg.v7.5.1.symbols.gmt” gene set file from the Gene Set Enrichment Analysis (GSEA) website (https://www.gsea-msigdb.org/gsea/msigdb/) as the predefined gene set. We applied stringent criteria to identify signaling pathways with significant enrichment: a normalized enrichment score (NES) with |NSE|>1, *p*<0.05, and a false discovery rate (FDR) <0.25. The GSEA was conducted on the DEGs using the ClusterProfiler package in R to uncover their collective involvement in biological processes and pathways.

### 2.5 Statistical analysis

Statistical analyses were conducted using R software. Categorical variables were presented as numbers and percentages [n (%)]. Continuous data not conforming to a normal distribution were reported as medians with interquartile ranges (IQRs). The Mann-Whitney U test was utilized for comparative analyses between two groups. The chi-square test or the corrected chi-square test was applied to assess differences in the distribution of frequencies and percentages. Binary logistic regression was employed to identify independent risk factors associated with disease severity and in-hospital outcomes. The diagnostic performance of these risk factors was assessed using the receiver operating characteristic (ROC) curves and the area under the curve (AUC). The Youden index, derived from the ROC curve, was used to determine the optimal threshold for risk indicators, facilitating the reclassification of severe patients into different subgroups.

Additionally, Kaplan-Meier survival analysis and log-rank test were used to compare the 30-day survival rates. The date of admission was defined as the starting point of the observation period, with the termination point being the date of death. A *p*-value of less than 0.05 was considered statistically significant.

## 3 Results

### 3.1 Basic characteristics of elderly COVID-19 patients

A total of 281 COVID-19 patients were enrolled in this study, including 158 males (56.23%) and 123 females (43.77%), with a median age of 75.00 years. Among these patients, hypertension (57.65%) and diabetes (33.45%) were the most prevalent comorbidities. Based on the clinical status at admission, 212 patients (75.44%) with mild or moderate COVID-19 were categorized into the non-severe group and 69 patients (24.56%) with severe or critical cases were categorized into the severe group. A minimum of 46.26% (130/281) of the participants were not vaccinated, while at least 29.54% (83/281) had received three or more vaccine doses. Notably, no statistically significant differences were observed in vaccination dose between participants with non-severe and severe COVID-19 cases (*p*>0.05), or between severe cases that resulted in survival versus fatality. This finding differs from the results of previous study (Alessandra et al., 2024) and may be associated with the relatively high proportion of unvaccinated elderly participants in our study. This discrepancy suggests that vaccination rates among the elderly could significantly influence the potential protective effect of vaccines on disease severity.

The median length of hospitalization for patients with non-severe COVID-19 was 11 days (IQR 8-15), contrasting with the 18-day median for those with severe COVID-19 (IQR: 12-28). According to in-hospital outcomes, severe patients were further classified into 49 severe survivors (71.01%) and 20 severe non-survivors (28.99%), as shown in **Table 1**. The survivor subgroup exhibited a median hospital stay of 19 days (IQR: 13.5-29.5), in contrast to the non-survivor subgroup, which had a median stay of 9 days (IQR: 7.5-19.5). Statistical analysis revealed a significant difference in the length of hospitalization between the survivor and non-survivor groups. Conversely, the distribution comorbidities did not exhibit statistically significant variations between these two groups.

**Table 1:** Clinical characteristics of elderly COVID-19 patients.

Variables	Non-severe(n=212)	severe(n=69)	*p*	survivor(n=49)	Non- survivor(n=20)	*p*
**Sex**			0.083			0.594
**male**	113 (53.30%)	45 (65.22%)		31 (63.27%)	14 (70.00%)	
**female**	99 (46.70%)	24 (34.78%)		18 (36.73%)	6 (30.00%)	
**Age**	74.00 (68.00, 83.00)	76.00 (70.50, 84.00)	0.062	76.00 (70.00, 82.50)	81.00 (72.00, 85.00)	0.29
**Length of hospitalization**	11.00 (8.00, 15.00)	18.00 (12.00, 28.00)	<0.001*	19.00 (13.50, 29.50)	9.00 (7.5, 19.50)	0.008*
Comorbidities						
**Hypertension**	116 (54.72%)	46 (66.67%)	0.081	32 (65.31%)	14 (70.00%)	0.707
**Diabetes**	72 (33.96%)	22 (31.88%)	0.751	17 (34.69%)	5 (25.00%)	0.433
**Chronic cardiovascular disease**	67 (31.60%)	22 (31.88%)	0.965	17 (34.69%)	5 (25.00%)	0.433
**Chronic kidney disease**	30 (14.15%)	6 (8.70%)	0.239	4 (8.16%)	2 (10.00%)	1.000
**Tumor**	31 (14.62%)	9 (13.04%)	0.744	8 (16.33%)	1 (5.00%)	0.382
**Vaccination**			0.316			0.401
0	95 (44.81%)	35 (50.72%)		25 (51.02%)	10 (50.00%)	
1-2	29 (13.68%)	9 (13.04%)		7 (14.29%)	2 (10.00%)	
≥3	68 (32.08%)	15 (21.74%)		12 (24.49%)	3 (15.00%)	
unknown	20 (9.43%)	10 (14.49%)		5 (10.20%)	5 (25.00%)	

*p<0.05.

### 3.2 Immunological characteristics upon hospital admission in elderly COVID-19 patients

An analysis was conducted to evaluate peripheral blood lymphocyte subset levels upon hospital admission. In comparison with the non-severe group, significant reductions were observed in counts of CD8^+^ T cells, CD4^+^ T cells, and NK cells among severe patients (all *p*<0.05, **Table 2**). Additionally, patients with severe COVID-19 exhibited a significantly reduced peripheral blood monocyte count, in contrast to increased levels of NLR, SAA and CRP, as compared to the non-severe group (all *p*<0.05, **Table 3**). These laboratory observations are consistent with previous research (Kang et al., 2024), indicating the activation of an inflammatory response. A comparative analysis between severe non-survivors and survivors revealed significantly higher levels of NLR and IL-6 in non-survivors (all *p*<0.05, **Table 3**). However, no significant differences were observed in the peripheral blood lymphocyte subset counts between these two groups (**Table 2**). Collectively, these results suggest that the immunological changes in elderly COVID-19 patients are closely associated with disease severity and clinical prognosis.

**Table 2:** Analysis of lymphocyte subset in elderly COVID-19 patients upon hospital admission.

Variables	Non-severe(n=212)	Severe(n=69)	*p*	survivor(n=49)	Non-survivor(n=20)	*p*
CD8^+^ T cell	195.00 (120.00, 309.75)	110.00 (60.00, 235.00)	<0.001*	120.00 (73.00, 235.00)	68.00 (30.50, 256.25)	0.197
CD4^+^ T cell	274.50 (154.25, 446.00)	123.00 (72.50, 259.50)	<0.001*	154.00 (76.50, 262.50)	98.50 (69.50, 197.50)	0.264
NK cell	137.00 (77.00, 254.50)	98.00 (45.00, 187.00)	0.008*	100.00 (63.00, 213.50)	83.00 (30.75, 174.25)	0.443
B cell	83.50 (45.00, 146.50)	70.00 (34.00, 131.00)	0.158	74.00 (38.00, 149.50)	36.00 (26.75, 111.25)	0.088

*p<0.05.

**Table 3:** Analysis of inflammatory cell and cytokine in COVID-19 patients upon hospital admission.

Variables	Non-severe(n=212)	Severe(n=69)	*p*	survivor(n=49)	Non-survivor(n=20)	*p*
Monocyte	0.42 (0.26, 0.58)	0.31 (0.22, 0.43)	0.005*	0.31 (0.15, 0.49)	0.23 (0.15, 0.38)	0.269
NLR	5.01 (2.84, 9.58)	11.83 (6.31, 21.28)	<0.001*	11.55 (5.84, 18.32)	18.23 (7.18, 37.58)	0.050*
CRP	43.80 (17.03, 97.68)	79.70 (38.15, 157.95)	<0.001*	73.70 (33.35, 141.40)	115.80 (58.08, 177.53)	0.059
SAA	288.85 (134.15, 398.13)	374.90 (274.81, 417.90)	0.007*	365.70 (211.65, 411.70)	398.10 (334.68, 423.60)	0.065
IL-6	34.29 (13.08, 114.83)	55.26 (9.78, 291.17)	0.366	26.85 (7.75, 91.95)	264.12 (69.85, 472.35)	0.001*

*p<0.05.

### 3.3 Multivariate analysis of factors influencing the severity of COVID-19 and in-hospital outcomes

A binary logistic regression analysis was conducted to evaluate the association between COVID-19 disease severity (categorized as non-severe=0 and severe=1), and the risk factors identified as statistically significant in the univariate analysis. These factors were considered as independent variables in the model. As shown in **Table 4**, NLR (odds ratio: 1.03 [1.001-1.060]) was an independent risk factor of disease exacerbation in elderly COVID-19 patients. Furthermore, a reduced CD4^+^ T cell count was found to be significantly and independently correlated with an increased risk of severe disease.

**Table 4:** Multivariate analysis of factors influencing the severity of COVID-19.

Variables	β	OR	95%CI	*p*
**CD4^+^ T cell**	-0.003	0.997	0.995-1.000	0.024*
**NLR**	0.03	1.03	1.001-1.060	0.04*

*p<0.05.

In the comparison between the severe survivor group and the severe non-survivor group, both the NLR and IL-6 demonstrated statistically significant differences. The logistic regression analysis showed that NLR (odds ratio: 1.039 [1.000-1.078]) was an independent risk factor, significantly influencing the in-hospital outcomes among severe patients (**Table 5**).

**Table 5:** Multivariate analysis of factors influencing the in-hospital outcomes in severe COVID-19 patients.

Variables	β	OR	95%CI	*P*
**IL-6**	0.002	1.002	1.000-1.003	0.055
**NLR**	0.038	1.039	1.000-1.078	0.047*

*p<0.05.

**Figure 1:**
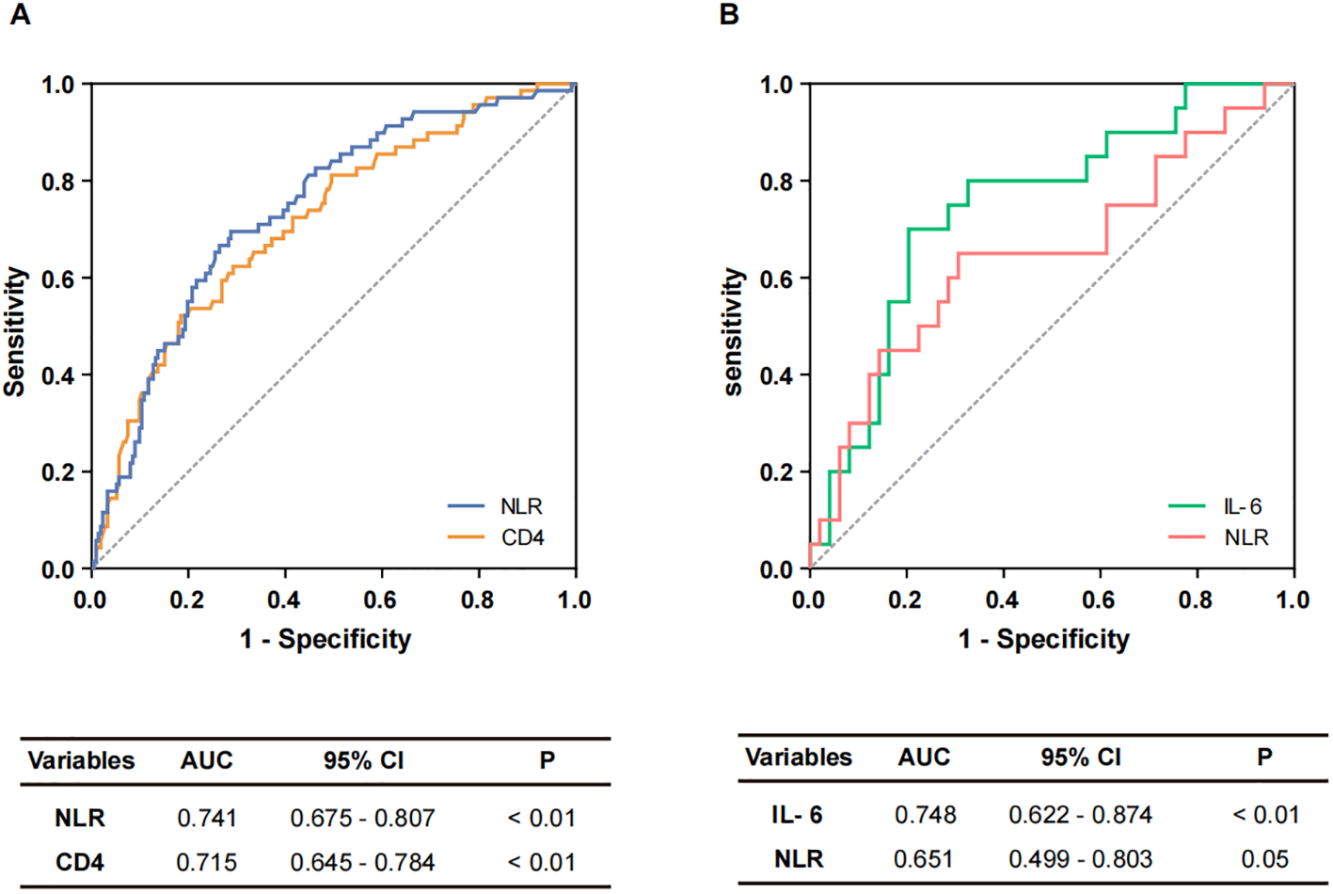
The diagnostic performance of potential markers. **(A)** Potential markers of diagnostic value to distinguish non-severe and severe cases. ROC curves of NLR and CD4^+^ T cells. **(B)** Potential markers of diagnostic value for distinguishing non-survivors from survivors. ROC curves of IL-6 and NLR.

Subsequently, the diagnostic efficacy of the identified independent risk factors for differentiating between non-severe and severe COVID-19 patients was evaluated. ROC curves and AUCs are shown in **Figure 1**. In the assessment of COVID-19 severity, the AUCs for CD4^+^ T cells and NLR were 0.715 (95% confidence interval [CI]: 0.645-0.784) and 0.741 (95% CI: 0.675-0.807), respectively (**Figure 1A**). A logistic regression-based diagnostic model, which included both risk factors, resulted in an AUC of 0.728 (95% CI: 0.660-0.796), indicating no superiority over the NLR alone. Moreover, the AUC for NLR in distinguishing severe survivors from non-survivors was 0.651 (95% CI: 0.499-0.803). Given the clinical significance of IL-6, an evaluation of its diagnostic potential for predicting in-hospital outcomes was also undertaken. The AUC for IL-6 in identifying severe non-survivors was 0.748 (95% CI: 0.622-0.874) (**Figure 1B**). In a combined diagnostic model established by logistic regression analysis, the AUC for the joint detection of NLR and IL-6 was 0.730 (95% CI: 0.584-0.876), which did not exceed that of IL-6 alone.

### 3.4 Prognostic value of immunological indicators on admission day

**Figure 2:**
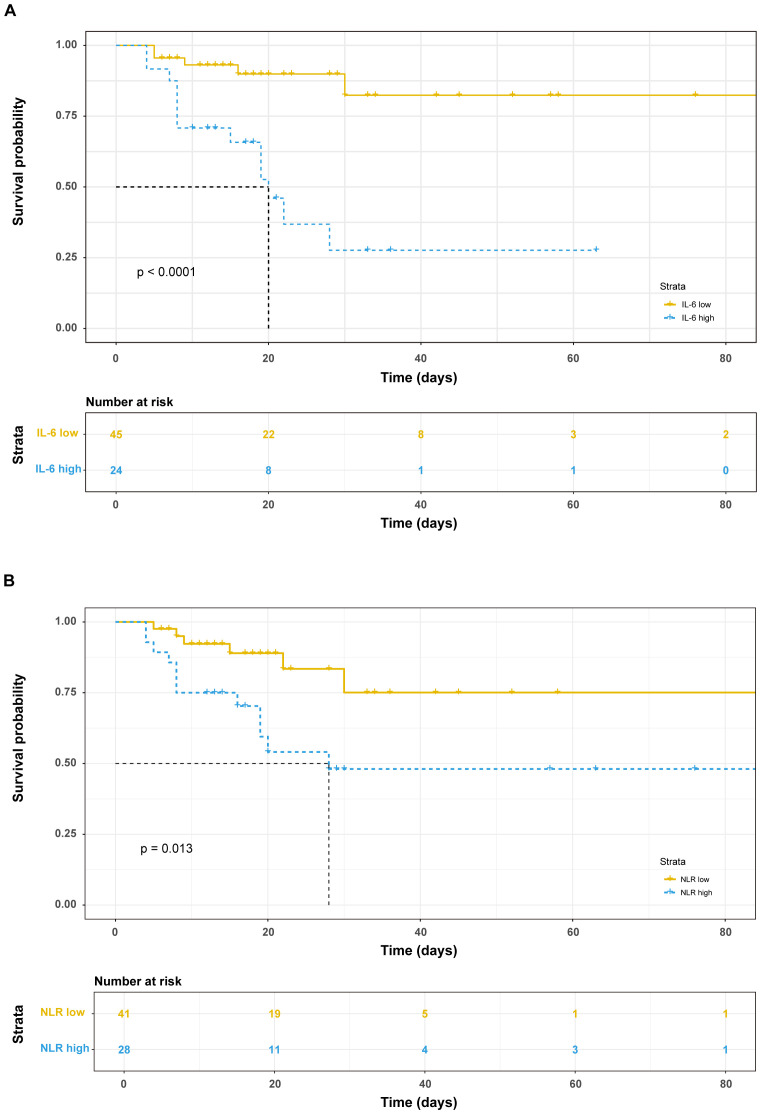
Prognostic value of immunological factors on admission day. Kaplan-Meier survival analysis and the log-rank test were performed in IL-6 **(A)** and NLR **(B)**.

Among the 69 patients with severe COVID-19, 18 fatalities were recorded within a 30-day period following admission. Employing the Youden index from the ROC curve, the optimal cut-off values for IL-6 and NLR to distinguish between survivors and non-survivors were determined to be 113.2 and 15.94, respectively. Subsequently, patients were stratified into subgroups based on these thresholds: IL-6 <113.2 pg/ml and >113.2 pg/ml groups, as well as NLR <15.94 group and NLR >15.94 group. Survival analysis revealed that patients with elevated IL-6 levels (>113.2 pg/ml) or high NLR (>15.94) exhibited significantly reduced survival times (**Figure 2**).

### 3.5 Acquisition of single-cell transcriptome data

Given the marked reduction in CD4^+^ T cell levels among elderly patients with severe COVID-19, we conducted a single-cell transcriptome analysis to elucidate the underlying molecular mechanisms related to these clinical characteristics. Initially, we accessed the GSE165080 dataset from the GEO database. Then, we extracted data from individuals aged 60 years or older from the “Asymptomatic (AS)”, “Moderate (MD)”, and “Severe (SD)” groups and merged them into a new dataset. This newly formed dataset consisted of ten elderly patients with severe COVID-19 (median age: 69.00 years) and six elderly patients with non-severe COVID-19 (median age: 64.50 years).

**Figure 3:**
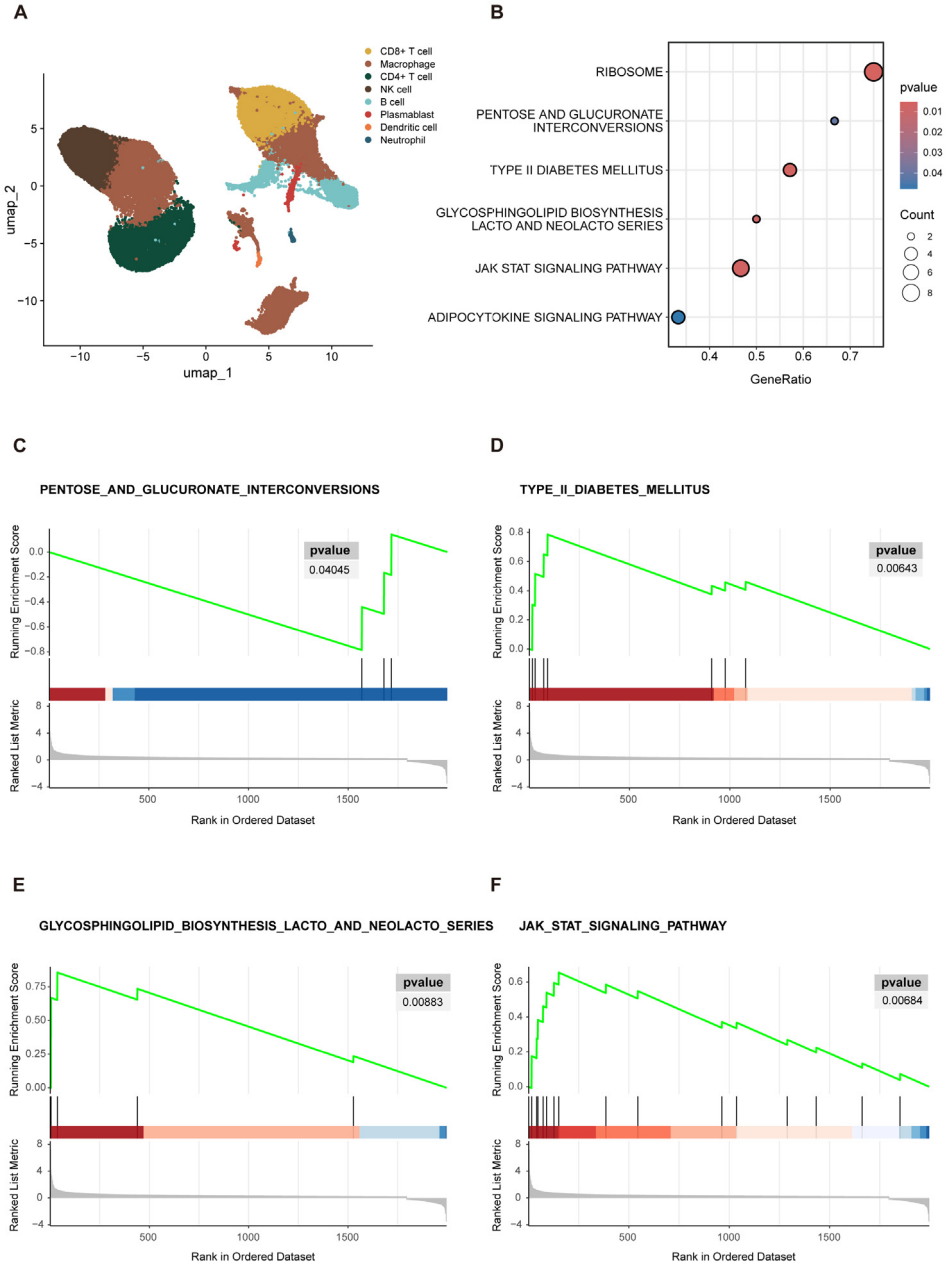
GSEA visualization results. **(A)** UMAP projections of single-cell sequencing data, captured from 16 COVID-19 patients and were annotated by cell type. **(B)** Enrichment bubble plot. The Y-axis denoted the descriptive information of the enriched pathways. “GeneRatio” was the proportion of differentially expressed genes (DEGs) in a specific pathway compared to the total number of DEGs. “Count” represented the number of DEGs in the enriched pathways. The size of the circles in bubble chart represented the number of genes enriched in the corresponding pathways. Larger circles indicated a higher number of enriched genes. The color of the circles reflected significance of the enrichment. A deeper shade of red indicated a lower *P*-value, indicating a higher level of significance for the pathway enrichment. **(C–F)** The GSEA analysis was depicted in three distinct sections. The uppermost segment was the Enrichment Score (ES) plot, which reflected the degree of gene set enrichment. The central component was a line plot that marked the position of each gene within the gene set. The third section, depicted as a gray butterfly plot, which represented the gene-phenotype association matrix and illustrated the distribution of ranked values of all genes. PENTOSE AND GLUCURONATE INTERCONVERSIONS **(C)** showed downregulation in the severe group, as indicated by a negative ES. In contrast, TYPE II DIABETES MELLITUS **(D)** GLYCOSPHINGOLIPID BIOSYNTHESIS LACTO AND NEOLACTO SERIES **(E)**, and JAK STAT signaling pathway **(F)** exhibited upregulation in the severe group, as evidenced by positive ES.

Following this, we conducted preliminary categorization and annotation of peripheral blood cells in the newly acquired dataset. The main cell lineages identified included B cells, CD4^+^ T cells, CD8^+^ T cells, macrophage, NK cells, and other cell types (**Figure 3A**). To further investigate the functional characteristics of the CD4^+^ T cells cluster, we specifically extracted the CD4^+^ T cells data and performed GSEA analysis to compare the “SD” group with the combined “AS+MD” group to identify molecular pathways and biological processes correlated with disease severity.

### 3.6 Gene Set Enrichment Analysis

The results of GSEA revealed several biological pathways significantly correlated with the severity of COVID-19 in elderly patients. Specifically, 6 signaling pathways showed substantial enrichment, including the RIBOSOME, PENTOSE AND GLUCURONATE INTERCONVERSIONS, TYPE II DIABETES MELLITUS (T2DM), GLYCOSPHINGOLIPID BIOSYNTHESIS LACTO AND NEOLACTO SERIES, JAK STAT signaling pathway, and ADIPOCYTOKINE signaling pathway (**Figure 3B**). These enriched pathways highlighted the multifaceted biological disruptions potentially instigated by severe COVID-19 in the elderly, with several pathways playing pivotal roles in orchestrating immune responses. Viruses are dependent on the ribosomes of host cells to facilitate replication and protein synthesis, both of which are essential for the viral life cycle and pathogenicity. Thus, the RIBOSOME pathway is critical in SARS-CoV-2 infection and was notably enriched in the GSEA results presented in this study. In addition to the RIBOSOME pathway, the gene set contributed most significantly to PENTOSE AND GLUCURONATE INTERCONVERSIONS (**Figure 3C**), with core genes mainly including UGP2 and DCXR, the enrichment score (ES) of this pathway was -0.786. Negative scores denote a downregulation of pathways, which may imply metabolism compromise in elderly patients with severe COVID-19. In contrast, an upregulation pattern was observed in the T2DM (**Figure 3D**), GLYCOSPHINGOLIPID BIOSYNTHESIS LACTO AND NEOLACTO SERIES (**Figure 3E**), JAK STAT signaling (**Figure 3F**), and ADIPOCYTOKINE signaling pathway, with an ES > 05.

## 4 Discussion

After implementing the “zero-COVID” policy for more than two years, China made significant adjustments to its pandemic response strategies on December 7, 2022 (Xinhua, 2022). This shift has been accompanied by a notable escalation in the number of infections, particularly among the elderly population, where there has been a significant concurrent increase in severe cases (Xiao et al., 2023). Previous research has indicated that age is a critical factor that may affect the accurate interpretation of study outcomes (Molani et al., 2022), emphasizing the importance of conducting separate analyses for different age cohorts. In light of these considerations, this study focused on elderly COVID-19 patients with the aim to establish a scientific foundation for the early identification of risk factors and the enhancement of clinical outcomes for these patients.

Compared with non-severe patients, a statistically significant reduction in CD4^+^ T cells, CD8^+^ T cells, NK cells, and monocytes was observed in patients with severe COVID-19 (*p*<0.05). This finding aligns with previous studies that have underscored the pivotal role of inflammatory markers in peripheral blood as indicators for assessing the disease severity (Kang et al., 2024). Monocytes, serving as immune response cells during the initial phase of infection, are susceptible to pyroptosis following SARS-CoV-2 invasion, which in turn releases potent inflammatory signals. In severe cases, the observed reduction in monocyte levels correlates with a concurrent elevation in inflammatory markers, including CRP, SAA, and NLR. The NLR is recognized as a reliable and independent prognostic factor for disease severity, with elevated levels indicating an increased risk of systemic inflammation and the potential for multi-organ failure in elderly patients with COVID-19 (Mathew et al., 2020). Patients with increased IL-6 levels (>113.2 pg/ml) or elevated NLR (>15.94) exhibited reduced survival rates and shorter survival times within 30 days after admission. Conversely, lower IL-6 and NLR levels are suggestive of effective disease management, while higher levels may correlate with disease progression and adverse prognosis (Rosas et al., 2021).

The recruited monocytes release a variety of cytokines that stimulate the activation and differentiation of T and B cells, particularly the T cell-mediated immune response, which has a broader cross-reactivity and longevity (Sette et al., 2023). Histological examination of pulmonary tissue from COVID-19 fatalities has revealed interstitial mononuclear inflammatory infiltration, predominantly composed of lymphocytes (Xu et al., 2020). The co-occurrence of lymphopenia alongside elevated CRP levels is indicative of a cytokine storm, a severe form of immune dysregulation characterized by excessive inflammation. Moreover, the exhaustion of T cells induced by SARS-CoV-2 infection significantly impairs cellular immunity (He et al., 2021). In severe COVID-19 cases, significant perturbations in peripheral T cell homeostasis and function have been observed. The phenotype of CD4^+^ T cells varied according to the severity of the disease. Severe patients exhibited a redistribution of CD4^+^ T cell subsets, including loss of naïve T cells, skewing towards a TH2-like immune response, and the emergence of a central memory T (TCM) cluster with a type I IFN-responsive signature (Mathew et al., 2020; Notarbartolo et al., 2021). Additionally, there was an expansion of CD4^+^ T follicular helper cells, cytotoxic CD4^+^ T cells, and terminally differentiated, exhausted T cells (Adamo et al., 2021). Importantly, CD4^+^ T cells have been identified as an independent factor influencing disease progression in elderly COVID-19 patients.

We utilized publicly accessible single-cell sequencing dataset to explore the underlying biological processes of CD4 T^+^ cells in elderly individuals with COVID-19. Firstly, the investigation unveiled that macrophages and T cells accounted for a substantial proportion of all cell types (**Figure 3A**), signifying their abundance in the peripheral blood of elderly COVID-19 patients (Sefik et al., 2022; Tay et al., 2020). Then, GSEA revealed 6 biological pathways associated with disease severity. Although these findings require validation through further experimental studies, the collectively enriched pathways suggest that CD4^+^ T cells are under considerable survival stress. Drawing on the primary biological functions of these pathways, it is plausible to infer that hyperinflammation and metabolic dysregulation are hallmark features of the COVID-19 pathophysiology in the elderly.

Specifically, the JAK/STAT signaling pathway serves as a common pathway and an effector molecule for numerous cytokines, playing a central role in COVID-19-related hyperinflammation (Yang et al., 2021). Recent studies have demonstrated that the IL-6-JAK-STAT3 axis is closely associated with the severity of COVID-19 (Giamarellos-Bourboulis et al., 2020; Hirano and Murakami, 2020). Moreover, JAK inhibitors such as ruxolitinib, have been proven to be safe and effective in treating COVID-19 patients with defined hyperinflammation (Hammersen et al., 2023). Our investigation further revealed that the JAK/STAT signaling pathway was upregulated in severe elderly COVID-19 patients, suggesting a disease severity-dependent augmentation in inflammatory signaling. Underlying conditions such as diabetes and obesity, along with impaired metabolism, are recognized as predominant host risk factors contributing to the development of severe complications associated with SARS-CoV-2 infection (Conte et al., 2024; Jiang et al., 2021). We observed a functional imbalance in the T2DM and the PENTOSE AND GLUCURONATE INTERCONVERSIONS pathway, indicating a potential disruption in energy metabolism among elderly patients. It is noteworthy that the incidence of T2DM has risen following COVID-19, with a higher increase observed among hospitalized patients compared to non-hospitalized patients, and the duration of the condition appears to be prolonged. The risk of type 2 diabetes is heightened with the severity of COVID-19. In contrast, this increase in incidence is less apparent in individuals who have been vaccinated against COVID-19 (Taylor et al., 2024). The relationship between diabetes (primarily type 2) and COVID-19 is bidirectional, as not only both conditions are significant risk factors for acute and chronic manifestations, but there is an association between COVID-19 and the incidence of diabetes. Furthermore, other pathways, such as the ADIPOCYTOKINE signaling pathway, play a pivotal role in various critical physiological processes, including energy metabolism and the regulation of inflammatory responses.

Metabolism and immune response are inextricably linked (O’Neill et al., 2016). Comprehensive analysis of immune cell subpopulations is instrumental in deepening our comprehension of metabolic regulation, particularly in relation to the specific release of cytokines and the distinction between proinflammatory and immunosuppressive roles among diverse immune cell types. Our results suggest that immunological profiles serve as predictive markers for COVID-19 severity and mortality. The observed reduction in CD4^+^ T cell counts in elderly patients is detrimental to disease recovery, and the pathways modulated by these cells may increase susceptibility to SARS-CoV-2 infection, potentially exacerbating the pathogenesis of associated complications. It is crucial to proactively screen individuals with a history of COVID-19 for metabolic diseases and to further investigate the pathological mechanisms and targeted therapeutic strategies for COVID-19-associated metabolic disorders.

Our study showcases several strengths, but we acknowledge some limitations. Firstly, it was conducted in a specific geographic region, which may limit the generalizability of our findings. Additionally, patients infected with various sub-variants during the Omicron wave might exhibit different base level of immunological characteristic in peripheral circulation. Future research should consider larger and more diverse cohorts to address these limitations, and emphasize the importance of longitudinal studies to track immunological changes over time in response to various SARS-CoV-2 variants.

## Data Availability

The original contributions presented in the study are publicly available. This data can be found here: https://doi.org/10.6084/m9.figshare.27634152.
